# Healthy lifestyle practice correlates with decreased obesity prevalence in individuals with high polygenic risk: TMM CommCohort study

**DOI:** 10.1038/s10038-024-01280-3

**Published:** 2024-08-22

**Authors:** Yoichi Sutoh, Tsuyoshi Hachiya, Yayoi Otsuka-Yamasaki, Shohei Komaki, Shiori Minabe, Hideki Ohmomo, Makoto Sasaki, Atsushi Shimizu

**Affiliations:** 1 Division of Biomedical Information Analysis, Iwate Tohoku Medical Megabank Organization, Disaster Reconstruction Center, Iwate Medical University, Yahaba, Japan; 2 Division of Biomedical Information Analysis, Institute for Biomedical Sciences, Iwate Medical University, Yahaba, Japan; 3 Iwate Tohoku Medical Megabank Organization, Disaster Reconstruction Center, Iwate Medical University, Yahaba, Japan; 4 Division of Ultrahigh Field MRI, Institute for Biomedical Sciences, Iwate Medical University, Yahaba, Japan

**Keywords:** Risk factors, Genome-wide association studies

## Abstract

Obesity and overweight, fundamental components of the metabolic syndrome, predispose individuals to lifestyle-related diseases. The extent to which adopting healthy lifestyles can reduce obesity risk, even in those with a high genetic risk, remains uncertain. Our aim was to assess the extent to which lifestyle modifications can improve outcomes in individuals with a high polygenic score (PGS) for obesity. We quantified the genetic risk of obesity using PGSs. Four datasets from the Tohoku Medical Megabank Community-Based Cohort (TMM CommCohort) were employed in the study. One dataset (*n* = 9958) was used to select the best model for calculating PGS. The remaining datasets (total *n* = 69,341) were used in a meta-analysis to validate the model and to evaluate associated risks. The odds ratio (OR) for obesity risk in the intermediate (11th–90th percentiles in the dataset) and high PGS categories (91st–100th) was 2.27 [95% confidence intervals: 2.12–2.44] and 4.83 [4.45–5.25], respectively, compared to that in the low PGS category (1st–10th). Trend analysis showed that an increase in leisure-time physical activity was significantly associated with reduced obesity risk across all genetic risk categories, representing an OR of 0.9 [0.87–0.94] even among individuals in the high PGS category. Similarly, sodium intake displayed a positive association with obesity across all genetic risk categories, yielding an OR of 1.24 [1.17–1.31] in the high PGS category. The risk of obesity was linked to the adoption of healthy lifestyles, even in individuals with high PGS. Our results may provide perspectives for integrating PGSs into preventive medicine.

## Introduction

Obesity and overweight are integral components of metabolic syndrome, which increases the risk of developing various lifestyle-related diseases, including type 2 diabetes [1–4]. The global prevalence of obesity in adults ≥18 years old exceeded 650 million in 2016 and has approximately tripled since 1975 [5]. Ongoing global initiatives are aimed at mitigating the rising prevalence of obesity.

For prevention, transitioning to a healthier lifestyle is widely recommended. Simultaneously, susceptibility to obesity is influenced by lifestyle and heritable factors. Recent whole-genome sequencing analyses suggest a heritability estimate for body mass index (BMI) between 0.28 and 0.30 (standard error = 0.10) [6]. The use of polygenic scores (PGSs) has been developed as a method to quantitatively represent the genetic risk in an individual [7]. However, a fundamental question remains: to what extent can an individual with a high PGS for obesity improve outcomes through lifestyle modifications? Although previous studies have explored the impact of lifestyle changes on individuals with varying genetic risks for coronary disease [8], comprehensive evidence across various phenotypes is lacking to evaluate the practical efficacy of PGS.

In this study, we analyzed one of the largest cross-sectional datasets with genotype information from a Japanese community cohort, aiming to provide insights into this inquiry. We determined the optimal model for calculating PGS to predict dichotomized obesity status, rather than directly using non-dichotomized BMI, to simplify the interpretation of obesity risk and facilitate practical application in future clinical settings. Our results could offer valuable viewpoints for the lifestyle considerations in the imminent era of precision medicine tailored to genetic risk profiles.

## Materials and methods

### Study population

The design for the Tohoku Medical Megabank Community-Based Cohort (TMM CommCohort) study has been previously described [9, 10]. Four datasets from the cohort were used in the present study: TMM10K, TMM67K, TMM18K, and TMM8K (Table 1). TMM10K and TMM67K included the participants recruited at specific health checkups, while TMM18K and TMM8K comprised those who visited the assessment centers for the cohort study. The overlapping samples between the datasets were retained in TMM10K and excluded from other datasets. We utilized the TMM10K dataset to identify the best model to predict obesity, and the other three datasets, TMM67K, TMM18K, and TMM8K, for the subsequent analysis.

**Table 1:** Characteristics of the study population

	TMM10K (model selection)	TMM67K (validation)	TMM18K (validation)	TMM8K (validation)
n	9811	50195	11947	6796
Female, %	65.2	60.9	75.6	66.8
Age, years	60.6 ± 11.2	60.4 ± 11.3	58.0 ± 12.9	55.7 ± 13.5
BMI, kg/m^2^	23.6 ± 3.6	23.5 ± 3.6	22.8 ± 3.5	23.3 ± 3.6
Waist circumference, cm	82.7 ± 9.4 (29)	83.0 ± 9.4 (112)	83.2 ± 9.7 (38)	83.4 ± 9.7 (15)
VFA (CT), cm^2^	n.d.	n.d.	n.d.	59.5 ± 32.4 (77)
Body fat rate (BIA), %	n.d.	n.d.	29.1 ± 7.7 (1)	n.d.
Obesity (BMI), %	30.6	30	23.2	27.7
Obesity (Waist), %	28.1 (29)	29.6 (112)	30.1 (38)	32.3 (15)
Obesity (VFA), %	n.d.	n.d.	n.d.	11.5 (77)
Obesity (BIA), %	n.d.	n.d.	59.5 (1)	n.d.
LTE, MET-h/d	2.6 ± 4.0 (3191)	2.8 ± 4.2 (13116)	2.7 ± 3.8 (778)	2.0 ± 3.3 (269)
DLA, MET-h/d	22.6 ± 14.1 (3191)	23.5 ± 14.8 (13116)	20.1 ± 11.7(778)	21.9 ± 12.9 (269)
Sodium intake, g/d	4.1 ± 0.9 (13)	4.0 ± 0.9 (85)	3.8 ± 0.9 (10)	4.1 ± 0.9 (3)
Array	OEE	JPAv2	JPAv2	JPAv2
Residency^a^	Iwate and Miyagi	Iwate and Miyagi	Miyagi	Iwate
Recruited	Health checkup in local government	Health checkup in local government	Visited an assessment center in TMM	Visited an assessment center in TMM
Allele count in PGS estimation^b^	9996136	9989402	9991396	9987906
PGS [95%CI]^b,c,d^	–1.983 × 10^–7^ [–1.989 × 10^–7^ to –1.978 × 10^-7^]	–1.964 × 10^–7^ [–1.966 × 10^–7^ to –1.961 × 10^–7^]	–1.940 × 10^–7^ [–1.946 × 10^–7^ to –1.935 × 10^-7^]	–1.945 × 10^–7^ [–1.953 × 10^–7^ to –1.938 × 10^-7^]

Number in parenthesis indicates count of missing values

*TMM* Tohoku Medical Megabank, *BMI* body mass index, *VFA* visceral fat area, *CT* computed tomography, *BIA* bioelectrical impedance analysis, *LTE* leisure-time exercise, *DLA* daily life activities, *MET* metabolic equivalent, *OEE* OmniExpressExome array, *JPAv2* Japonica array version 2, *PGS* polygenic risk score, *CI* confidence intervals, *SE* standard error

^a^Prefecture in Japan

^b^Best model

^c^Before normalization

^d^95%CI: mean ± 1.96SE

Sodium intake was estimated via spot urine using Tanaka’s formula [11], as previously described [12]. Physical activity was estimated using self-administered questionnaires [13]. Leisure time exercise (LTE) includes slow walking, fast walking, and light to moderate and strenuous exercise. Daily life activity (DLA) includes occupational activity, such as sitting, standing, walking, and strenuous work.

This study adhered to the principles of the Declaration of Helsinki. Written informed consent was obtained from all participants. Approval was granted by the Institutional Review Board of Iwate Medical University (HG H25-2). Analyses involving personal genotype or phenotype information were performed on a standalone computing system, the Tohoku University Tohoku Medical Megabank Organization (ToMMo) supercomputer [14].

### Genotyping and imputation

Genotyping and genotype imputation were conducted as previously described [10, 15, 16]. Genotyped data were pre-phased using Eagle version 2.4.1 [17] and imputed using Minimac3 version 2.0.1 [18] with the East Asian subset from 1000 Genomes Phase 3 [19] as the reference panel. Individuals were excluded from the analysis if they had a low call rate (<0.95), non-Japanese ancestry, or a sex mismatch between genotype and cohort data. Variants with a low call rate (<0.95), low Hardy–Weinberg equilibrium exact test *p*-value (*p* < 1 × 10^–6^), low minor allele frequency (<0.01), or low-imputation quality (*R*^*2*^ < 0.3) were also excluded. The allele counts subsequent to quality control are summarized in Table 1.

### PGS

To formulate a polygenic model, we estimated the weights of single nucleotide polymorphisms using publicly available genome-wide association study (GWAS) summaries derived from 158,284 Japanese individuals in BioBank Japan [20]. Variants characterized by low imputation quality (*R*^*2*^ < 0.3) were excluded from the GWAS summary. Two methods were used for modeling: linkage disequilibrium (LD) pruning plus thresholding (P + T) and LDpred (version 1.0.11) [21]. For the P + T method, 24 models were generated under different LD-pruning conditions using six *p*-value thresholds (1, 5 × 10^–1^, 5 × 10^–2^, 5 × 10^–4^, 5 × 10^–6^, and 5 × 10^–8^) and four *R*^*2*^ value thresholds (0.2, 0.4, 0.6, and 0.8). For LDpred, seven models were formulated for each *ρ* value (denoting the fraction of causal variants; *ρ* = 1, 0.3, 0.1, 0.03, 0.01, 0.003, and 0.001). Individual PGSs were computed using Plink2-v2.00a2LM [22, 23] for each model. PGSs were individually normalized for each dataset (TMM67K, TMM18K, TMM8K) with the mean and standard deviation of each.

### Statistical analyses

Statistical analyses were performed using R version 4.2.1. The areas under the curve (AUCs) were assessed using “ROCR” in the R package (version 1.0.11) with adjustment for age and sex [24]. For AUCs with 95% confidence intervals (CIs), we used “pROC” in the R package (version 1.18.0) [25]. In this study, obesity was delineated as a BMI ≥ 25 kg/m^2^, consistent with the local definition in Japan [26]. A meta-analysis of the validation datasets was performed using the fixed-effect model with the R package “meta” (version 6.5-0). We also used the random-effect model when heterogeneity was significant (*p* < 0.05). The simulation-based power calculation was conducted using the R package ‘metapower’ (version 0.2.2) [27]. In the present study, we performed a meta-analysis to pool results in three validation datasets: TMM67K (*n* = 50,195), TMM18K (*n* = 11,947), and TMM8K (*n* = 6796), with a two-tailed test and a significance level (*p*) of 0.05. The power of these sample sizes and design was estimated under various conditions determined by the heterogeneity estimate value between datasets (*I*^2^: 0, 0.2, 0.4, 0.6, 0.8) and expected effect size (odds ratio: 1.01–1.08, by 0.01) based on the random-effects model.

## Results

### Construction of a polygenic model for obesity risk

**Fig. 1:**
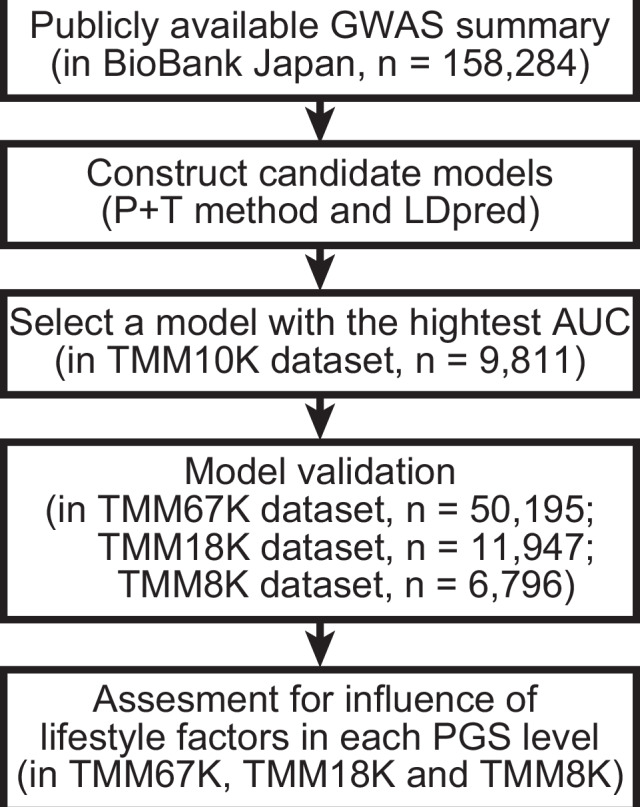
Flowchart of the study. Schematic of the study progression. See Table 1 for the characteristics of each dataset. PGS polygenic score, AUC area under the curve, TMM Tohoku Medical Megabank

**Fig. 2:**
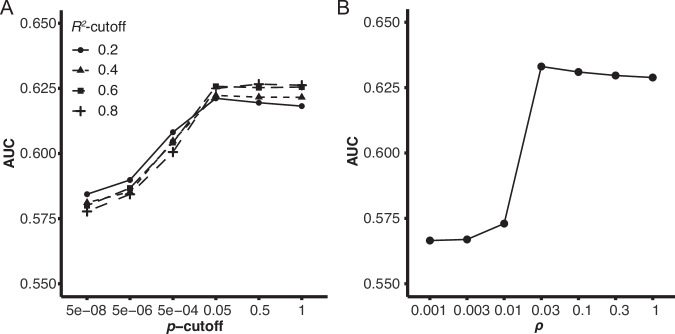
Model selection. Models for estimating the PGS were constructed employing either the linkage disequilibrium pruning and thresholding method (**A**) or LDpred [21] (**B**), using the effect sizes of variants in a publicly available genome-wide association study summary for BMI [20]. Individual PGS was ascertained using candidate models constructed within the TMM10K dataset. The AUC for obesity (BMI ≥ 25) was assessed in R package “ROCR,” with age and sex as covariates. The LDpred model at *ρ* = 0.03 exhibited the highest AUC and was used in subsequent analyses. PGS polygenic score, AUC area under the curve, BMI body mass index, TMM Tohoku Medical Megabank

A flowchart of the study is represented in Fig. 1. Owing to limited choices for calculating PGSs in the East Asian population, a new model was constructed for this study. In the TMM10K training dataset, the PGS determined by the model created using LDpred at *ρ* = 0.03 demonstrated the most optimal AUC amongst the evaluated models (Fig. 2). After adjusting for sex and age, the PGS for predicting obesity yielded an AUC of 0.63. Additionally, the PGS from this model indicated the highest correlation coefficient in those by candidate models (Spearman’s *ρ* = 0.246) with continuous BMI, under transformation and adjustment by age, age^2^, sex, and the first 10 principal components (Fig. S1) [20]. We compared this AUC with those of recently published models validated by datasets containing individuals of East Asian descent (Table S1). Our model showed AUCs of 0.62 [95% CI: 0.61–0.63] in the R package “pROC.” The models reported by Privé et al. (PGS002161) and Tanigawa et al. (PGS001228) showed AUCs of 0.61 [0.60–0.62] and 0.60 [0.59–0.62], respectively, within the TMM10K dataset. This implies that our current model performs similarly in estimating obesity risk, closely aligning with these recent models.

### Validation

**Fig. 3:**
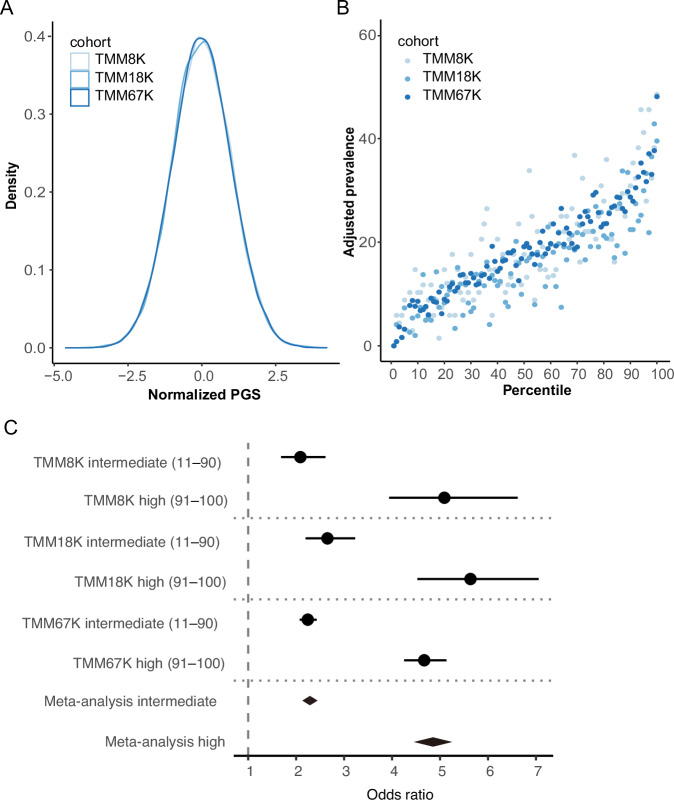
Model validation. Normalized PGSs in three validation datasets exhibited a similar distribution (*p* > 0.05 in the F-test comparing the distribution of each dataset to the pool of all validation samples) (**A**). The obesity prevalence, which was adjusted to the baseline (1st percentile) prevalence of zero, increased with elevation of the PGS percentile (**B**). The OR for obesity risk was determined by the low polygenic risk group (percentile 1–10) as reference (**C**). The fixed effect model was used for the meta-analysis. The error bars indicate the 95% confidence interval. OR odds ratio, PGS polygenic score, TMM Tohoku Medical Megabank

We determined PGSs in the validation datasets (TMM67K, TMM18K, and TMM8K) using the previously mentioned best-performing model (Fig. 3). The normalized PGS distributions across datasets were statistically analogous (Fig. 3A; *p* = 0.9859 in the Kruskal–Wallis test). The prevalence of obesity corresponding to each PGS percentile was calculated. To adjust for baseline variations in obesity prevalence across datasets, the prevalence in the 1^st^ (the lowest) percentile was subtracted from that in each percentile (Table S2) and depicted in a scatter plot (Fig. 3B). Moreover, the prevalence elevation curve remained consistent across all three datasets, exhibiting a sharp increase at both the lowest and highest ends of the curve compared with that of the middle. The center (50^th^) and top (100^th^) percentiles registered an augmentation of 17.6–19.1% and 39.5–48.5%, respectively, from the baseline (1st) percentile.

Guided by the prevalence curve, individuals were categorized into three risk groups for further analysis: low (1st to 10th percentile), intermediate (11th to 90th percentile), and high genetic risks (91st to 100th percentile). Subsequently, ORs were computed for both the intermediate and high genetic risk groups against the low genetic risk group as a reference (Fig. 3C). In a meta-analysis collating the outcomes from the three validation datasets, the intermediate and high genetic risk categories demonstrated obesity risks of 2.27 [95% CI: 2.12–2.44] and 4.83 [4.45–5.25], respectively.

Beyond the conventional definition of obesity based on BMI, there exist several other criteria employing different measures. To ascertain the robustness of our model in predicting obesity under these varied criteria, we conducted an evaluation using alternative definitions. The consistency and effectiveness of our model were determined by computing the AUC for obesity, as characterized by diverse parameters, including waist circumference, body fat percentage (BFP) derived from bioelectrical impedance analysis, and visceral fat area (VFA) ascertained using computed tomography (Table S3). The PGSs exhibited consistent AUC values (0.58–0.63) for obesity across these varied criteria. Additionally, we computed Spearman’s rank correlation coefficients (*ρ*) to assess the association between the PGS and various quantitative obesity-related metrics, including BMI, BFP, VFA, and waist circumference, each subject to rank-based inverse-normal transformation (Fig. S2). Notably, the PGS was significantly correlated (*p* < 0.05) with these quantitative metrics; the magnitude of association was modest (*ρ*: 0.131–0.201 in TMM67K, TMM18K, and TMM8K datasets) compared to that between PGS and BMI (*ρ*: 0.242–0.254). These results implied that our PGSs are associated with obesity defined by body shape (BMI and waist circumference) and measurements of body fat (BFP and VFA).

### Obesity risk and its relation to lifestyle factors based on genetic risk categories

**Fig. 4:**
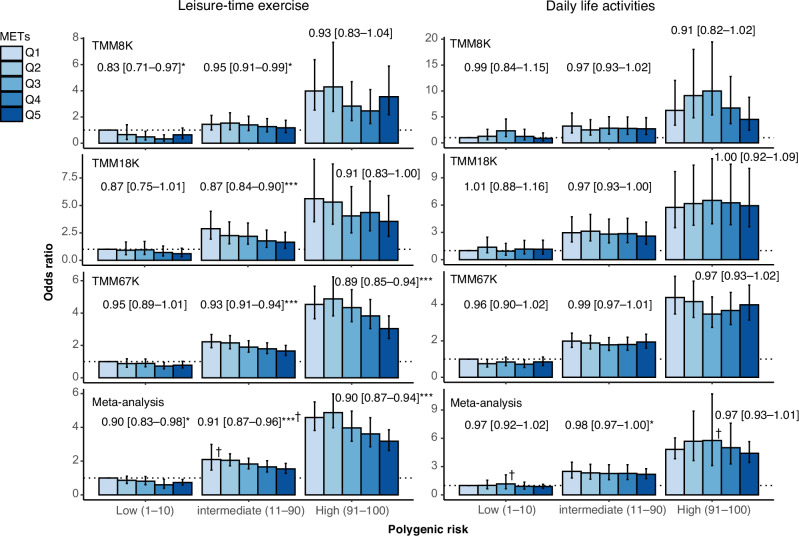
Effect of physical activity across different PGS stratifications. Obesity risk was evaluated in relation to physical activity across different PGS stratifications. The activity level was derived from a self-administered questionnaire and stratified into quintiles (Q1–Q5). Odds ratios (ORs) were calculated by logistic regression with adjustments for age and sex. The ORs in trend analysis are depicted above the bars, accompanied by a 95% confidence interval in square brackets. To account for the detected heterogeneity between cohorts (†, *p* < 0.05), the random effects model was employed for meta-analysis. The error bars indicate 95% confidence interval. PGS polygenic score, TMM Tohoku Medical Megabank, MET metabolic equivalent. * *p* < 0.05; ** *p* < 0.005, *** *p* < 0.0005

Physical activity [28, 29] and sodium intake [30, 31] are lifestyle factors that are associated with obesity risk. We evaluated the influence of these factors on obesity risk within different genetic risk categories. Regarding physical activity, obesity risk across quintiles of LTE and routine DLA was assessed (Fig. 4 and Table S4). In the meta-analysis of validation datasets, an increase in LTE quintiles was significantly correlated (*p* < 0.05) with reduced obesity risk (Fig. 4, left panel). Notably, the ORs in trend analysis were similar across all genetic risk strata (approximately 0.9 for each incremental increase of the LTE quintile). In contrast, DLA did not display a significant correlation with obesity risk in any of the genetic risk categories (Fig. 4, right panel).

**Fig. 5:**
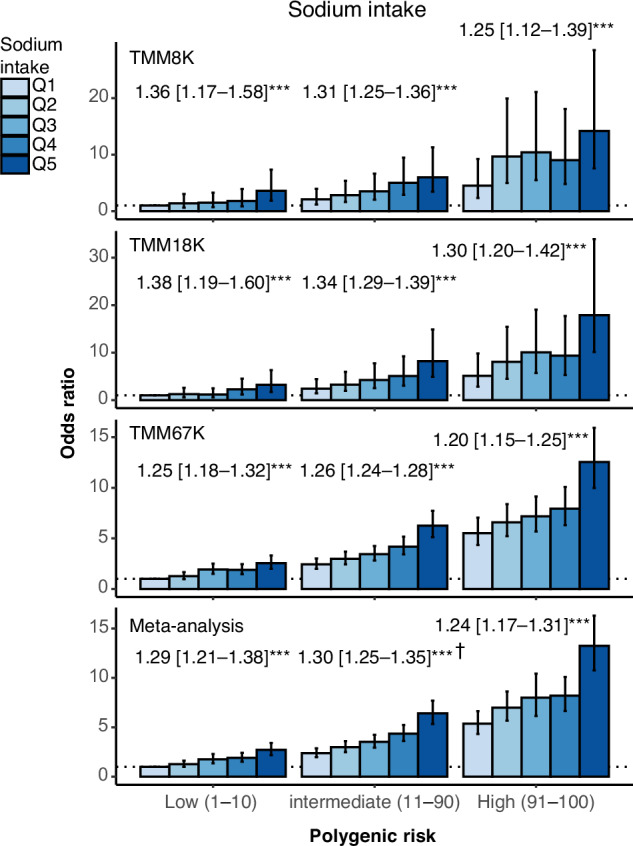
Effect of sodium intake across different PGS stratifications. Obesity risk was evaluated in relation to sodium intake across different PGS stratifications. Sodium intake, inferred from spot urine samples using Tanaka’s formula, was stratified into quintiles (Q1–Q5). Odds ratios (ORs) were calculated using logistic regression with adjustments for age and sex. Refer to the legend of Fig. 4 for comprehensive information

Regarding dietary habits, sodium intake, assessed via spot urine samples, demonstrated a significant correlation with obesity risk (Fig. 5). In a meta-analysis trend test, similar ORs of 1.29 [95% CI: 1.21–1.38], 1.30 [1.25–1.35], and 1.24 [1.17–1.31] were observed in the low, intermediate, and high genetic risk groups, respectively, for each incremental increase in sodium intake quintile.

To investigate potential sex-specific effects, we conducted stratified analyses of obesity risk by sex (Figs. S3–S6). This sensitivity analysis produced outcomes congruent with the main analysis. Notably, an incremental elevation in DLA was associated with a modest but statistically significant (*p* < 0.05) decrement in obesity risk among males with medium to high polygenic risk.

Furthermore, to elucidate the limitations of the study design, we calculated the statistical power of the meta-analysis under various odds ratios and heterogeneity (Fig. S7). To evaluate the impact of potential PGS deviation between datasets on the conclusions, we replicated our main analysis with the PGS normalized after pooling all validation datasets (TMM8K, TMM18K, and TMM67K) as a sensitivity analysis. Tables S5–S7 correspond to the analysis represented in Fig. 3C, Fig. 4 (LTE), and Fig. 5, respectively.

## Discussion

This study demonstrates the effect of LTE intensity on obesity risk across all genetic risk categories. The 5th quintile of LTE was >4.7, 4.5, and 3.1 MET-h/d for the TMM67K, TMM18K, and TMM8K datasets, respectively. As outlined in the Physical Activity Guidelines for Americans [32], activities spanning 3.0 to 6.0 METs are considered moderate-intensity endeavors and include brisk walking, doubles tennis, or yard raking. Moderate-to-vigorous LTE reduced both BMI and overall mortality rates [28]. Although DLA showed no clear correlation with obesity risk, our results suggested that the decline in obesity risk may result from environmental factors associated with LTE rather than from physical activity itself. Nevertheless, our findings indicated the potential of lifestyle improvements in mitigating obesity, even in individuals with a high genetic risk.

However, our results, although not optimistic regarding the preventive effect of LTE on obesity, revealed that obesity prevalence, when adjusted for cohort-specific background factors, was increased from 39.5 to 48.5% as one progresses from the baseline (1st) to the pinnacle (100th) of the PGS percentile, indicating the scale of genetic influence on obesity risk. Notably, even among individuals within the highest quintile of LTE, the risk of obesity remained marginally or significantly higher in those who were in the high genetic risk group than in those in the intermediate or lower genetic risk groups, respectively. This underscores the difficulty of mitigating genetic risk through acquired factors. Therefore, from a public health perspective, especially in individuals with a high genetic risk of obesity, optimizing prevention programs may be a valuable approach.

Moreover, we confirmed an association between obesity risk and sodium intake across all genetic risk tiers. Despite previous studies suggesting that this association is not influenced by energy consumption [30, 31], the underlying mechanism remains unclear. Further investigation is required to establish the causal relationship in this association and to explore potential strategies for future prevention.

A novel aspect of our research lies in elucidating the relationship between lifestyle factors and PGS for obesity, a comprehensive risk factor for multiple lifestyle diseases. Previous studies have focused on the association between favorable lifestyles and PGS for specific diseases, such as cardiovascular diseases [8, 33–36], dementia [37], breast cancer [38], lung cancer [39], and type 2 diabetes [40]. To the best of our knowledge, in the perspective of association with healthy lifestyles, there have been limited investigations regarding PGS of comprehensive risk factors such as blood pressure [41]. In prevention, these intermediate risk factors, easily monitored at home and suitable for setting health goals, prove useful. Nevertheless, these factors are also influenced by genetic factors [20, 42]. Hence, our current study could be instrumental in establishing a personalized weight management plan, considering genetic predispositions.

BFP and VFA are distinct measurements obtained from BMI and waist circumference, focusing on the quantification of body fat. Notably, VFA is obtained as clinical data through direct visualization of fat using computed tomography. In the present study, our model’s PGS demonstrated significant correlations with various obesity-related metrics, including BMI, BFP, VFA, and waist circumference. Furthermore, the PGS demonstrated predictive accuracy with significant AUC values for obesity, as defined not only by BMI but also using these varied measures. These findings indicate the versatility and robustness of our model, suggesting its potential for broad application across different obesity definitions.

The AUC achieved by our model for obesity prediction was 0.62 [95% CI: 0.61–0.63]. Comparative models by Privé et al. (PGS002161) and Tanigawa et al. (PGS001228) reported AUCs of 0.61 [0.60–0.62] and 0.60 [0.59–0.62], respectively, within the TMM10K dataset. Additionally, the observed correlation coefficient for the PGS in our study ranged from *r* = 0.245 to 0.263 for continuous BMI, as indicated in Fig. S2, without surpassing the accuracy of previous models. The GWAS sample size, from which the PGS model is derived, is a critical determinant of PGS accuracy [43]. For instance, Privé’s model (PGS002161), informed by a GWAS of 391,124 Europeans, achieved a correlation coefficient of *r* = 0.3664 [0.3544–0.3784] (Metric ID: PPM012135 in the PGS Catalog). This sample size was ~2.47 times larger than that in the Japanese GWAS, which we used for model construction. Although the method for model construction and number of effective variants for PGS calculation also affect accuracy, expanding the GWAS sample size could be a fundamental approach to enhance the predictive performance of PGS models.

This study had several limitations. First, the study is based on a community cohort restricted to a specific geographical area. Therefore, our results might not have excluded the influence of the unmeasured bias related to this limitation. Replication and validation in different populations are required. Next, the study used a cross-sectional dataset. This suggested the need for future longitudinal investigations to estimate the predictive performance of the PGS. Moreover, finally, we normalized the PGS in each dataset. This method could bias risk estimations if PGS distributions deviated significantly between datasets, although the sensitivity analysis suggests that the deviation had a limited impact on the conclusions of the present study.

In conclusion, we have developed PGS for obesity risk prediction and identified its associations with physical activity and sodium consumption. These insights might contribute to the development of effective and individualized prevention programs for obesity.

## Supplementary information

Figure S1-S7

Table S1

Table S2

Table S3

Table S4

Table S5

Table S6

Table S7

## Data Availability

The datasets of individuals in the TMM CommCohort study analyzed here are not publicly available owing to ethical reasons, such as protecting personal information and preventing unintended personal identification. However, they are accessible upon request, subject to approval from the Ethics Committee of Iwate Medical University and the Materials and Information Distribution Review Committee of the TMM Project. Those interested in obtaining the datasets for individuals may contact the corresponding author.
