# Supplementary material for: Healthy lifestyle practice correlates with decreased obesity prevalence in individuals with high polygenic risk: TMM CommCohort study
Source: J Hum Genet. 2024 Aug 22;70(1):9–15. doi: 10.1038/s10038-024-01280-3 (PMC11700849; doi:10.1038/s10038-024-01280-3)
Supplement: Supplementary file 1 — Figure S1-S7 [file 10038_2024_1280_MOESM1_ESM.pdf]

Supplementary figures

Figure S1

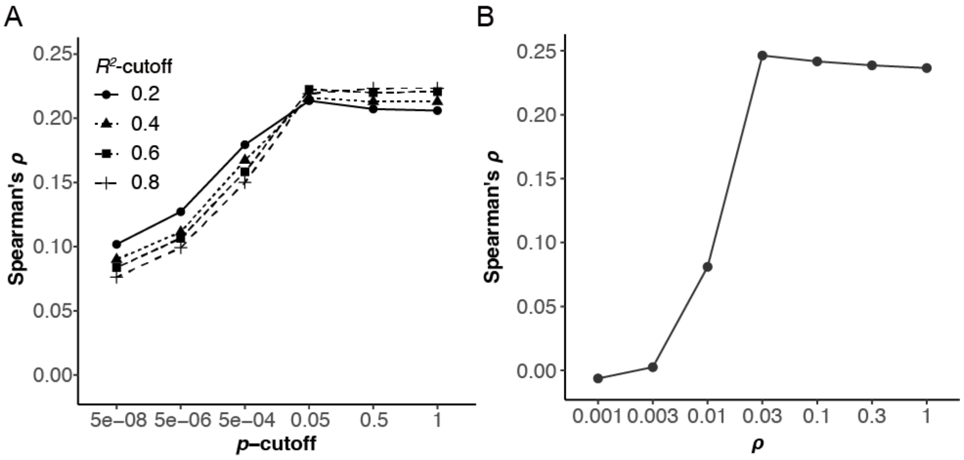

Figure S1. Correlation coefficient between continuous BMI and the PGS calculated by candidate models. The analysis illustrated in Figure 2 was conducted with continuous BMI rather than dichotomized obesity. The models were constructed using either the linkage disequilibrium pruning and thresholding method (A) or LDpred (B), respectively. We evaluated the correlation using Spearman's rank correlation coefficient (ρ). Prior to the correlation assessment, BMI values underwent a natural logarithmic transformation and adjustment for variables including age, age^2, sex, and the first 10 principal components (PC1-10). Subsequently, the residuals were subjected to a rank-based inverse-normal transformation (See ref. [20]). The best correlation was a Spearman's ρ = 0.246, achieved with the model by LDpred at parameter (ρ) = 0.03. BMI, body mass index; PGS, polygenic score

Figure S2

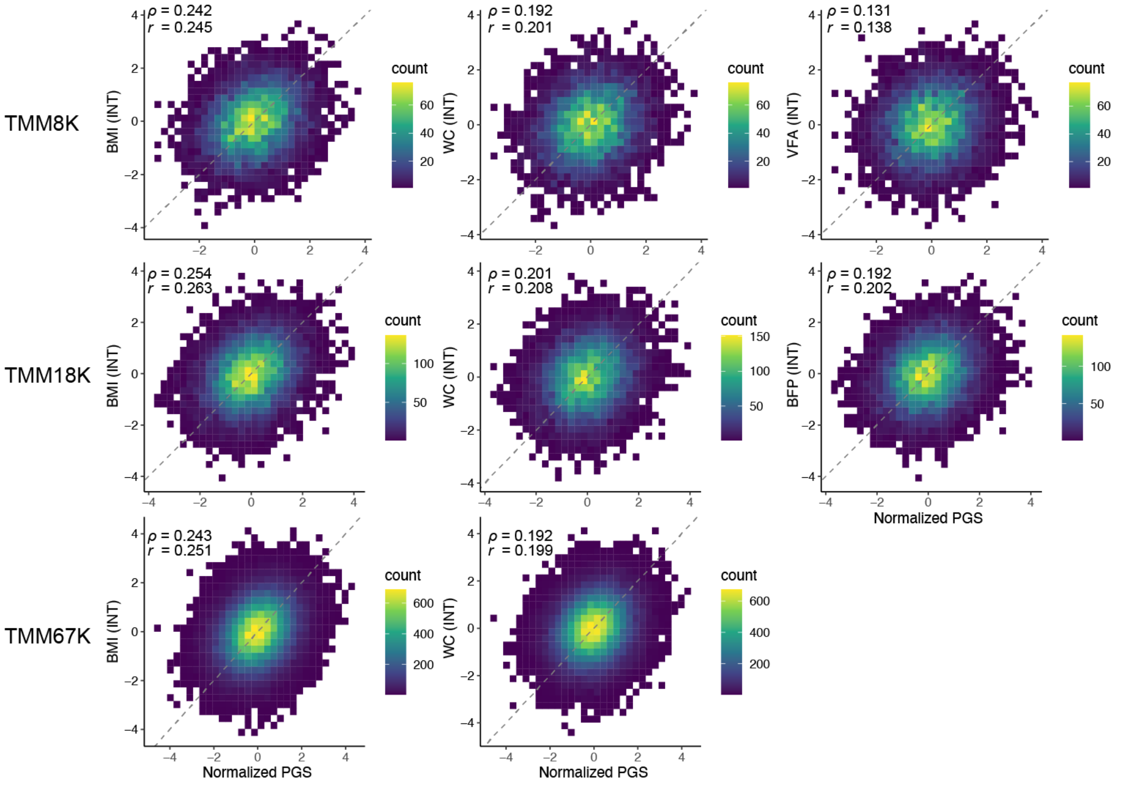

Figure S2. Correlation between PGS and various obesity-related measures. Before adjustment, BMI, visceral fat area (VFA), and body fat percentage (BFP) underwent a natural logarithmic transformation. The transformed values, along with the native values of waist circumference (WC), were adjusted for age, age^2, sex, and the initial 10 principal components (PC1-10). Subsequently, the residuals were processed using a rank-based inverse-normal transformation (INT). Correlation coefficients were calculated using both Spearman's rank correlation coefficient (ρ) and Pearson's correlation coefficient (r). All correlation coefficients presented here were statistically significant (p<0.05). The dotted line represents the diagonal. BMI, body mass index; PGS, polygenic score

Figure S3

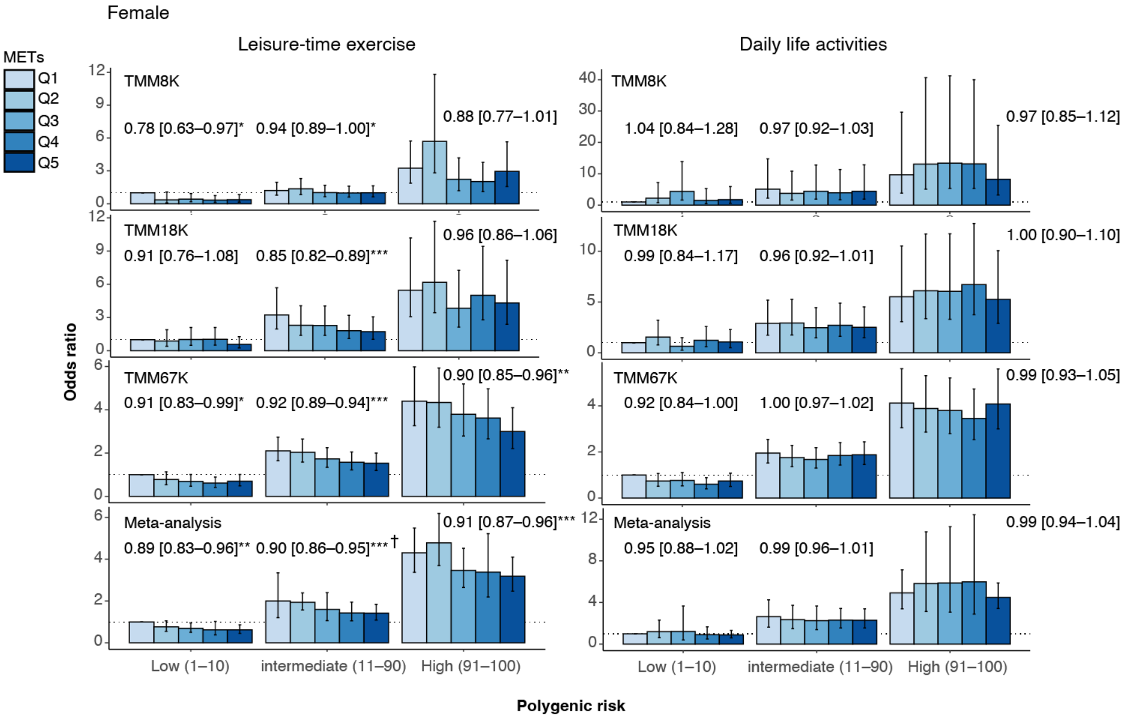

Figure S3. Effect of physical activity across different PGS stratifications (female only). Odds ratios were calculated using logistic regression with adjustments for age. Refer to the legend of Figure 4 for comprehensive information. PGS, polygenic score

Figure S4

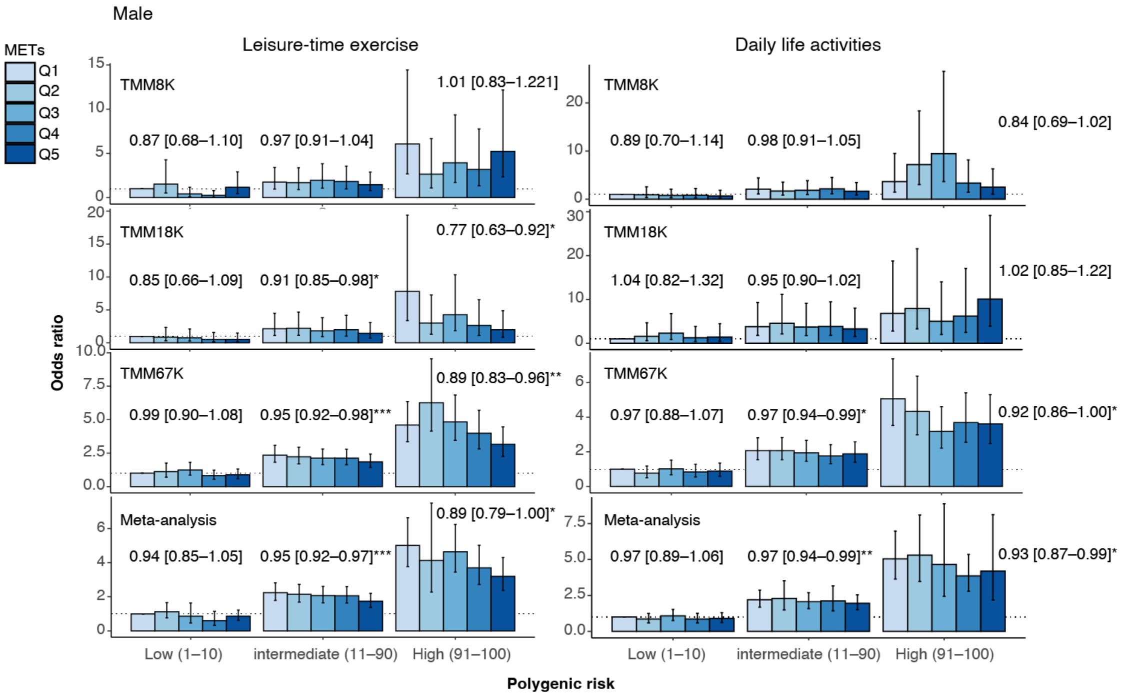

Figure S4. Effect of physical activity across different PGS stratifications (male only). Odds ratios were calculated using logistic regression with adjustments for age. See the legend of Figure 4 for comprehensive information. PGS, polygenic score

Figure S5

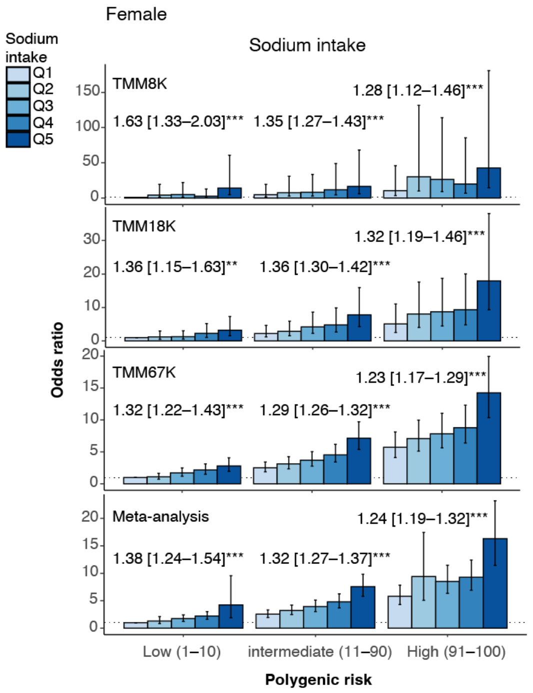

Figure S5. Effect of sodium intake across different PGS stratifications (female only). Odds ratios were calculated by logistic regression with adjustments for age. See the legend of Figure 4 for comprehensive information. PGS, polygenic score

Figure S6

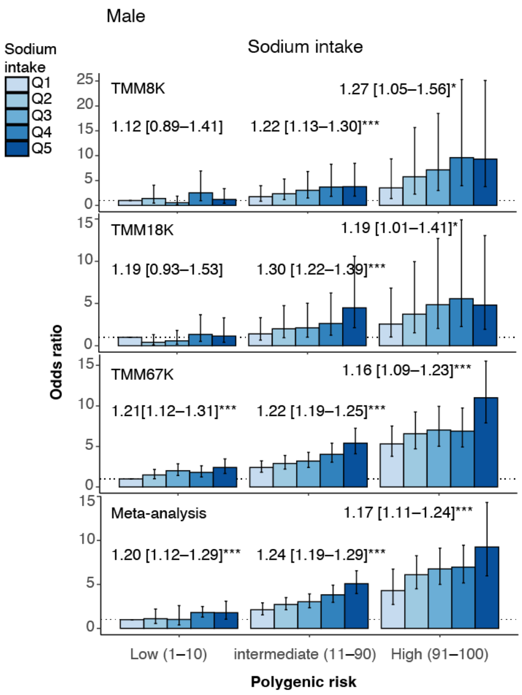

Figure S6. Effect of sodium intake across different PGS stratifications (male only). Odds ratios were calculated using logistic regression with adjustments for age. See the legend of Figure 4 for comprehensive information. PGS, polygenic score

Figure S7

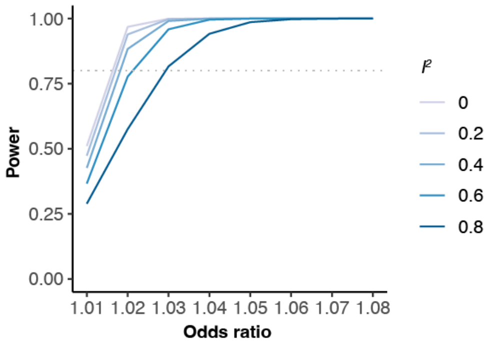

Figure S7. Power calculation for the current study design. The statistical power of the meta-analysis in the current study design (3 datasets, total n = 68,938) was estimated using the R package "metapower" (version 0.2.2) (https://cran.r-project.org/web/packages/metapower/index.html). The power was computed for a two-tailed test at a significance level of p=0.05 by employing a random-effects model with varying odds ratios and heterogeneity (I^2). The horizontal dashed line indicates a power of 0.8.
